# An Interesting Case of Neurobrucellosis Mimicking Neuropsychiatric Lupus

**DOI:** 10.1155/2018/9793535

**Published:** 2018-07-08

**Authors:** Ramandeep Bains, Tamara Dahhan, Annie Belzowski, Emil R. Heinze, Andrew L. Wong, Philip J. Clements

**Affiliations:** ^1^UCLA-Olive View Rheumatology Program, Division of Rheumatology, Olive View-UCLA Medical Center, 14445 Olive View Drive, 2B182, Sylmar, CA 91342, USA; ^2^UCLA-Olive View Internal Medicine Program, Department of Medicine, Olive View-UCLA Medical Center, 14445 Olive View Drive, 2B182, Sylmar, CA 91342, USA

## Abstract

This case describes a patient presenting with acute onset papilledema, subacute strokes resulting in upper extremity weakness and numbness, arthritis, maculopapular rash, depressed C4 and CH50, and a high titer anti-double-stranded DNA antibody. The patient was given the diagnosis of probable systemic lupus erythematosus, which was supported by the Systemic Lupus International Collaborating Clinics (SLICC) criteria. He was aggressively treated for neuropsychiatric lupus (NPSLE) with pulse dose steroids and a dose of intravenous cyclophosphamide. Blood cultures drawn on admission later grew out 2/4 bottles of Gram-variable bacteria, speciated as *Brucella melitensis* by PCR. Serum *Brucella* serologies were also positive. On further evaluation, the patient noted a history of eating unpasteurized cheese in Mexico. Given these additional findings, the patient's presentation was most consistent with a diagnosis of neurobrucellosis. Steroids were tapered off, no further doses of cyclophosphamide were given, and a prolonged course of intravenous and oral antibiotic therapy was administered, resulting in complete resolution of the patient's presenting symptoms.

## 1. Introduction

Brucellosis, secondary to *Brucella melitensis*, is the most common zoonotic infection in the world [1–4]. It is commonly seen in the Mediterranean Basin, Eastern Europe, South and Central America, Asia, Africa, the Middle East, and the Caribbean [1, 2, 4]. Brucellosis is considered to be eradicated in the United States since the early 1970s; however, sporadic cases of human infection in the US have occurred in the setting of consumption of unpasteurized dairy products from endemic countries [5]. Brucellosis is caused by a Gram-negative bacterium, *Brucella*, and is transmitted to humans by contact with infected animals and their bodily fluids or by consuming infected milk or milk products [1–3, 6–12]. The most common symptoms of human brucellosis infection include fever, headache, arthralgia, malaise, and sweating [2, 13, 14]. Brucellosis infection of the central nervous system (CNS) is a rare but serious complication [6, 15]. We report a case of neurobrucellosis mimicking the symptoms, laboratory data, and the pathologic findings that can be seen in systemic lupus erythematosus (SLE), thus demonstrating the diagnostic challenges of such a heterogeneous disease.

## 2. Case Presentation

A 50-year-old male with no past medical history presented to the hospital with one week of painless blurry vision of the right eye. He had also been having intermittent fevers, headache, body aches, and a nonpruritic maculopapular rash on the bilateral lower extremities for 6 months. On further review of systems, the patient noted one isolated episode of left knee swelling as well as testicular swelling in the past. The patient otherwise denied any neck stiffness, nausea, vomiting, Raynaud's phenomenon, oral ulcerations, chest pain, shortness of breath, abdominal pain, or photosensitivity. He worked as a flooring installer, and he did not have any toxic habits such as smoking, drinking, or illicit drug use.

**Figure 1:**
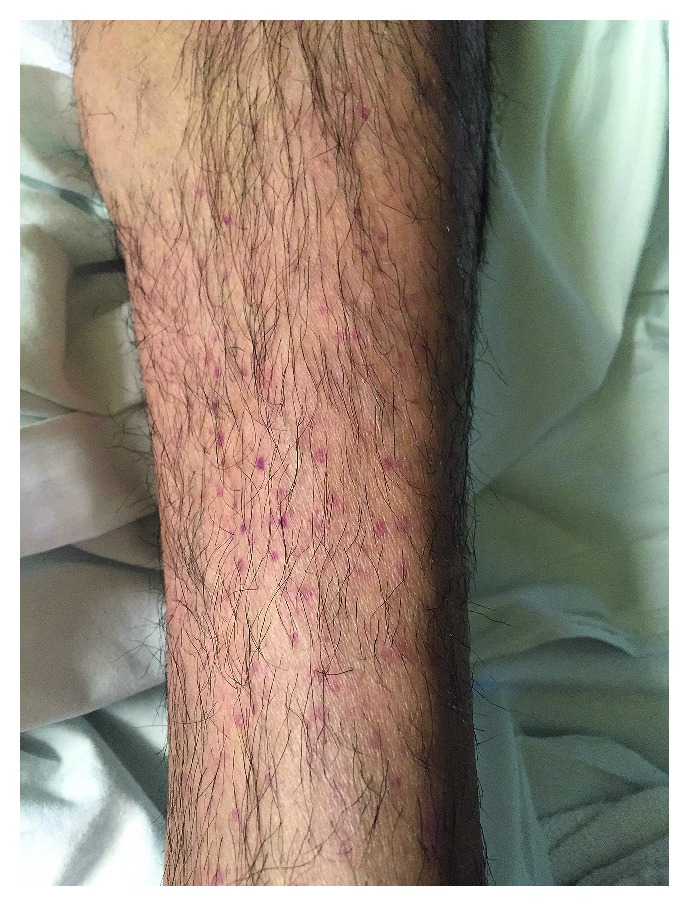


The patient's vital signs were normal. On physical exam, the patient was found to have bilateral papilledema and optic nerve erythema, right greater than left, right inferior nasal quadrant visual field defect, and a right afferent pupillary defect. Muscle strength was 5/5 throughout, and reflexes were 2+ throughout. Sensation to light touch, pinprick, vibration, and proprioception was intact. The bilateral lower extremities demonstrated a maculopapular rash (Figure 1).

The admitting labs were notable for a microcytic anemia (Hb 11.6 gm/dL (ref 13.6–17.3); Hct 35.3% (ref 39.8–50.7); MCV 76.9 fL (ref 80.3–98.1)), hyponatremia (133 mmol/L (ref 136–144)), elevated ESR (33 mm/hr (ref 0–15)), and elevated CRP (13.3 mg/L (ref 0.0–7.0)). Urinalysis did not show protein or blood. Lumbar puncture was colorless/clear with 2/cumm RBC (ref 0), 56/cumm WBC (ref 0–9), 39% segmented neutrophils (ref 0–2), 53% lymphocytes (ref 40–80%), 30 glucose (ref 40–70), 69 protein (ref 15–45), with presence of oligoclonal bands, an elevated IgG index (+19.8 mg/24 hr (ref −9.9 to +3.3)), and normal opening pressure (16 cm H20 (ref 10–25 cm H20)). The initial CT scan of brain and orbits demonstrated no acute intracranial process, and the MRI of the orbits was also unremarkable.

Given the patient's history of fever, myalgia, rash, and joint pain with CSF studies showing both a neutrophilic and lymphocytic pleocytosis, there was concern for infectious etiologies, including both bacterial and viral infections, as well as autoimmune etiologies. The differential diagnoses included neuromyelitis optica, multiple sclerosis, neuropsychiatric SLE, HIV, syphilis, tuberculosis, coccidioidomycosis, cryptococcus, Lyme, and West Nile virus.

Further investigation was pursued to work up the aforementioned etiologies, and the patient was found to have a positive double-stranded DNA (>1 : 640), low C4 (10 mg/dL (ref 16–47)), low CH50 (13 U/mL (ref 31–60)), normal C3, negative ANA by immunofluorescence assay (repeated twice) and negative anti-Sm/RNP, anti-SSA/B, Coombs antibody, anti-beta2 glycoprotein, anticardiolipin, and lupus anticoagulant. ANCA, ACE, and cryoglobulin were negative. Rheumatoid factor was positive (38 IU/mL (ref < 14)). The infectious disease service was consulted, and the infectious workup including HIV, hepatitis antibodies, cocci antibodies, RPR, cryptococcus antibodies, Lyme antibodies, West Nile virus antibodies, and Quantiferon Gold were all negative. CSF cultures showed no growth. Skin biopsy of the lower extremity rash was done, pending results.

The presence of a high-titer positive double-stranded DNA antibody raised concern for an autoimmune etiology, although in the setting of a negative ANA the validity of the dsDNA titer was questioned with a high concern for a false-positive test. Given the lack of other findings to suggest autoimmune disease, the rheumatology service requested additional studies. Given the negative infectious workup to date, the neurology service recommended initiation of pulse corticosteroids with methylprednisolone 1,000 mg intravenous daily for which the patient received 2 doses, with improvement in his symptoms, which was then followed by oral prednisone taper.

**Figure 2:**
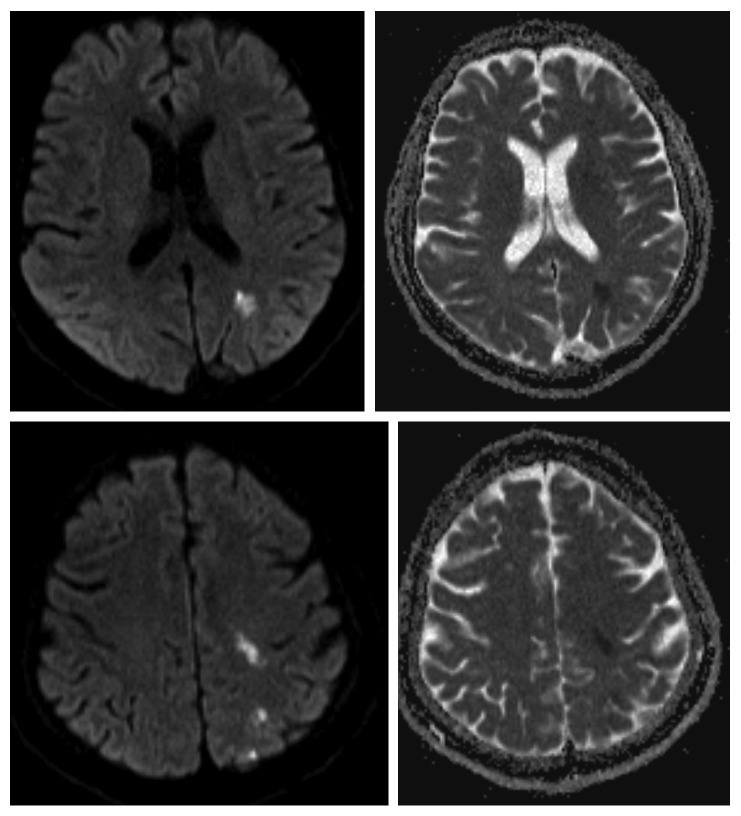


**Figure 3:**
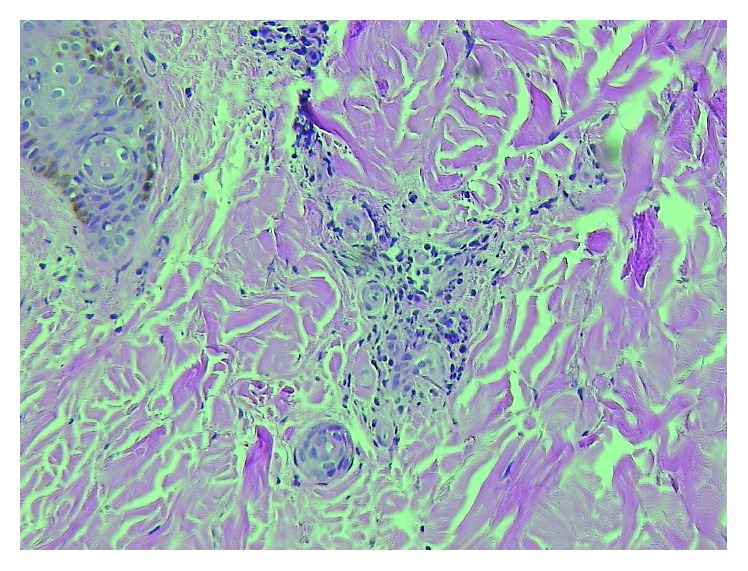


Approximately one week later, the patient returned to the hospital with acute onset right arm weakness and numbness. On physical exam, the patient was found to have 4/5 muscle strength in the right upper extremity with decreased sensation to light touch over the fourth and fifth digits of the right hand. MRI of the brain showed multiple subacute infarcts in the left parietal lobe (Figure 2). CT angiogram of the brain was negative. Furthermore, the results of skin biopsy had returned demonstrating leukocytoclastic vasculitis (Figure 3). With these new clinical, radiographic, and pathologic findings, there was concern for a CNS small vessel vasculitis possibly secondary to SLE given satisfaction of SLICC criteria which included low C4 and low CH50, high-titer double-stranded DNA, maculopapular rash of the lower extremities, and bilateral papilledema and erythema resulting from cranial neuropathy of the optic nerves. SLICC criteria require that there be greater than or equal to 4 criteria met including at least 1 clinical and 1 laboratory criteria. The patient's history of knee swelling also raised concern for synovitis, although this did not meet the SLICC criteria explanation for synovitis as our patient demonstrated swelling in only one joint, and SLICC criteria require synovitis in two or more joints. Other etiologies such as embolic phenomenon from endocarditis or paraneoplastic syndrome were considered; as a result, blood cultures were sent, and ECHO was done showing no valvular vegetations. Blood cultures had shown no growth × 5 days. As a result of the above workup, the patient was given a working diagnosis of neuropsychiatric SLE (NPSLE) with new onset neurologic changes, and he was treated with pulse dose methylprednisolone 1 gram for a total of five days with prednisone taper as well as one induction dose of intravenous cyclophosphamide 1,000 mg.

After the patient was discharged, the blood cultures that had been initially reported as no growth, were later found to have 2/4 bottles positive for Gram-variable bacteria. The cultures were sent to Public Health for confirmation, and the organism was speciated as *Brucella melitensis* by PCR. Subsequently, the patient was readmitted to the hospital and confirmed to have positive *Brucella* serologies including total antibody titer (1 : 320 (ref < 1 : 80)), IgG (6.99 (ref < 0.80)), and IgM (1.42 (ref < 0.80)). *Brucella* bacteremia, positive *Brucella* serum serologies, and clinical presentation with subacute stroke were all consistent with a diagnosis of neurobrucellosis. On further history, the patient noted to have eaten unpasteurized cheese in Mexico 6 months prior which was thought to be the source of infection. The corticosteroids were tapered off, no further doses of cyclophosphamide were given, and the patient was given four weeks of intravenous ceftriaxone as well as three months of oral doxycycline and rifampin.

On follow-up, the patient's serum *Brucella* IgM became negative, repeat blood cultures showed no growth, and repeat lumbar puncture demonstrated resolution of pleocytosis. The patient's symptoms of weakness, blurry vision, headaches, intermittent fevers, and body aches resolved. The patient's visual acuity returned to normal, and the papilledema resolved, but the patient was noted to have some residual optic nerve atrophy.

## 3. Discussion

Brucellosis is a multisystem disease with a wide variety of clinical manifestations making the diagnosis in nonendemic areas very challenging. Acute brucellosis manifestations are often nonspecific and can resemble other neurologic and rheumatologic diseases as demonstrated in the case above. Neurobrucellosis is a rare but serious complication of brucellosis infection, with an incidence that ranges between 0.5 and 25% [1, 6, 13, 15]. Neurobrucellosis also has a wide spectrum of clinical manifestation including both peripheral and central nervous system involvement. Peripheral manifestations tend to be more chronic while central manifestations tend to appear more acute [3]. Common manifestations of neurobrucellosis include meningitis, meningoencephalitis, myelitis, neuritis of a cranial or peripheral nerve, and/or vascular disease [6, 7, 10, 12, 16]. Pathogenesis is thought to be mediated by cytokine or endotoxin effect on neuronal tissues, cytotoxic T lymphocytes, and immunological mechanisms causing demyelinating lesions in the brain and spinal cord white matter [12].

The diagnosis of neurobrucellosis can be difficult given the diverse neurologic characteristics and lack of specific radiographic or serologic findings [1, 2, 10, 12]. Imaging abnormalities commonly seen are meningeal enhancement, white matter changes, and vasculitis [17]. Serologic testing is available but is only significant if interpreted in the presence of clinical findings compatible with brucellosis. A lymphocyte predominant pleocytosis of CSF has also been described in neurobrucellosis, as seen in our patient, although this is nonspecific and can be seen in many other infectious or inflammatory processes [1]. Culture of the organism is the gold standard to confirm diagnosis, but growth rate is slow and can lead to delay in diagnosis [7, 12]. Studies have demonstrated that the diagnosis of neurobrucellosis in most cases is usually made two to twelve months after the onset of symptoms. Neurobrucellosis has been documented to occur at any stage of the infection [10]. Our patient developed neurological symptoms long after what appeared to be his initial infectious exposure, six months earlier.

Neurobrucellosis manifesting as vasculitis, as seen in our patient, is an unusual but well-described manifestation of brucellosis [8, 12]. While studies of patients with neurobrucellosis have shown the most commonly affected cranial nerve is VIII [1, 10], and involvement of optic nerve secondary to vasculitis has been documented [10, 15, 18]. There are theories that these vasculitic changes may be related to an immune-mediated reaction in the CNS due to *Brucella* infection [17, 19].

Our patient's main complaint was visual and neurological symptoms, but the patient also gave an additional history of maculopapular rash on the lower extremities, intermittent fevers, headaches, body aches, an episode of left knee, and testicular swelling. All of these additional symptoms are well-described symptoms of acute brucellosis infection. In Burzgan et al.'s study of 1,028 patients with brucellosis, the most frequently seen symptoms in acute brucellosis were arthralgias (73.7%), myalgia (37.6%), headache (18.8%), and fever (76.9%). Scrotal swelling had an incidence of 3.8% and skin lesions 2.4% [2].

The presence of the high-titer positive dsDNA antibody described in the above case contributed to a much higher suspicion for SLE than would have otherwise been attributed to the case, thus highlighting the significance of the positive anti-dsDNA antibodies in delaying the correct diagnosis and treatment. Brucellosis-induced autoantibody production, has been described, as brucellosis has been shown to be a triggering factor for immunologic reaction [20]. The autoantibody level in the reticuloendothelial organs of brucellosis patients demonstrates the role of an autoimmune process in the pathogenesis of brucellosis. A study looking at the prevalence of autoimmune biomarkers in brucellosis patients by Ahmadinejad et al. found that patients with brucellosis have a higher rate of positive rheumatologic markers (i.e., ANA and rheumatoid factor) compared to the normal population [20]. Of the 49 patients with brucellosis tested for autoantibodies by Ahmadinejad et al., one patient was noted to have a positive dsDNA antibody, as well as a positive ANA [20]. Our patient was found to have positive double-stranded DNA as well as a positive rheumatoid factor, which may be been caused by brucellosis-induced autoantibody production.

While autoantibody production has been described in brucellosis patients, the possibility of lab error was also considered. Measurement of the dsDNA antibodies was done using the *Crithidia lucilia* immunofluorescence testing. This form of indirect immunofluorescence (IFA) testing is considered to have the highest specificity for detecting dsDNA antibodies, while the more common form of testing by ELISA (enzyme-linked immunosorbent assay) is considered to be less specific, but more sensitive [21]. The positive dsDNA antibodies coupled with the negative ANA by immunofluorescence was considered highly unusual, and the discordance between these tests is unclear. One possible explanation is a misinterpretation of the *Crithidia* IFA, as the reading of the test is operator dependent. *Crithidia lucilia* is a hemoflagellate protozoa that has an organelle called a kinetoplast which contains a high-concentration native double-stranded DNA. When IFA testing is used to test anti-dsDNA antibodies, the kinetoplast is the area which will fluoresce if these antibodies are present. The lab operator must correctly identify the kinetoplast's location, when compared to a control, although the exact location of this organelle may vary between organisms. If a different organelle stains positive for fluorescence, such as the basal body, this can introduce a risk for a true-negative test that is misinterpreted as positive. As a result, it is important that this test be interpreted with caution, especially in the setting of a negative ANA.

In conclusion, we present a diagnostically challenging case of an infection mimicking an autoimmune disease, thus highlighting the importance of ruling out infectious diseases when evaluating a patient with features of systemic vasculitis. This case also emphasizes the risk of associated production of autoantibodies in the setting of infection, as well as the risk of lab error and false-positive test results. While epidemiologic advances and cultural habits have resulted in low rates of brucellosis in the United States, the large movement of people and goods between the United States and endemic areas makes it increasingly important for healthcare providers to recognize brucellosis' diverse clinical presentations capable of mimicking other diseases.

## Data Availability

The case clinical data used to support the findings of this study are included within the article.
